# Management of a Large Bowel Obstruction Secondary to a 5cm Gallstone in a Rural Setting: A Case Report

**DOI:** 10.7759/cureus.78475

**Published:** 2025-02-04

**Authors:** Chenyi Mao, Jonathan Tiong, James Gallagher

**Affiliations:** 1 Department of Medical Education, The University of Melbourne, Melbourne, AUS; 2 Department of General Surgery, The Royal Melbourne Hospital, Melbourne, AUS; 3 Department of General Surgery, Wimmera Base Hospital, Horsham, AUS

**Keywords:** acute large-bowel obstruction, biliary ileus, cholecystectomy, cholecystocolic fistula, gallbladder disease

## Abstract

We present a case of a large bowel obstruction secondary to a large impacted gallstone measuring 5cm in the sigmoid colon. Significant features include a cholecysto-colonic fistula and the presence of a perforation initially missed on imaging. Endoscopic retrieval was attempted, followed by a Hartmann's procedure. This case highlights the significance of operative management in ensuring the safe treatment of colonic gallstone ileus, especially in a rural setting.

## Introduction

Gallstone ileus occurs in 0.3-0.5% of patients with cholelithiasis [1]. Gallstone sigmoid ileus accounts for 4% of all gallstone ileus patients [2]. Colonic obstruction from gallstone ileus tends to occur in elderly females with diverticular disease [3]. Although most reported colonic ileus has been treated surgically, the postoperative morbidity and mortality rates are high [3]. The 2022 census dictates that 28% of Australians reside in rural and remote Australia; however, only 12% of general surgeons live and work rurally, often serving populations across large geographical distances [4]. Hence, challenges exist for rural surgeons delivering emergency surgical care due to limited intensive care resources and staff for broader responsibility. We describe a case of colonic gallstone ileus, which was managed with both endoscopic and surgical methods.

## Case presentation

A 90-year-old woman presented to our emergency department in rural Australia with acute or chronic abdominal pain, vomiting, and constipation. Her background is significant for chronic cholecystitis managed with intravenous antibiotics three years ago. She has no other medical comorbidities and is independent of her activities of daily living. The patient had previously declined an elective cholecystectomy on account of her age. On presentation, her vital signs were within normal limits. The physical examination revealed a markedly distended abdomen with mild right-side abdominal tenderness.

Investigations

**Figure 1:**
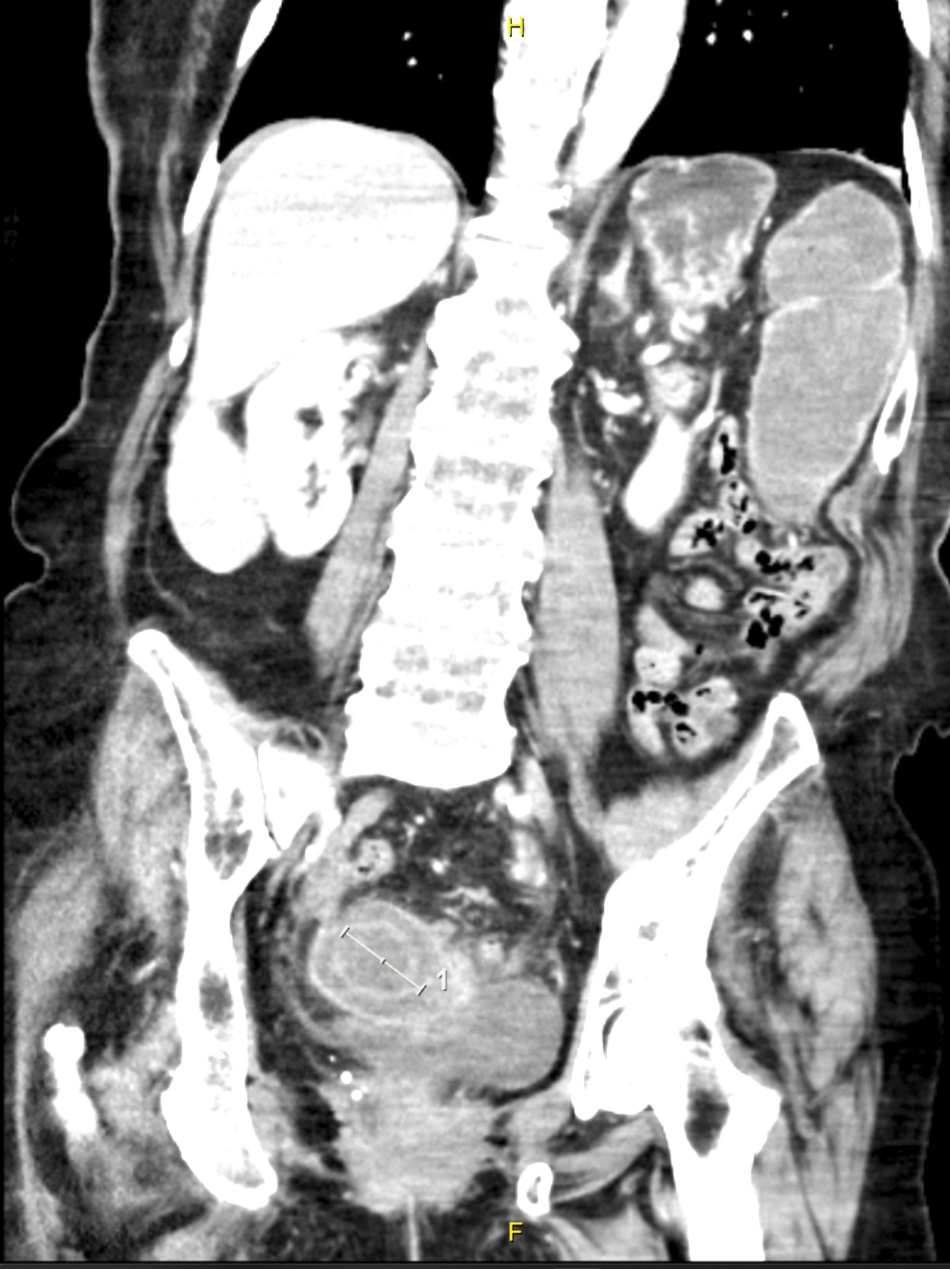
Abdominal CT scan: axial view showing a 34.74mm × 34.97mm gallbladder stone impacted in the mid to distal sigmoid colon

**Figure 2:**
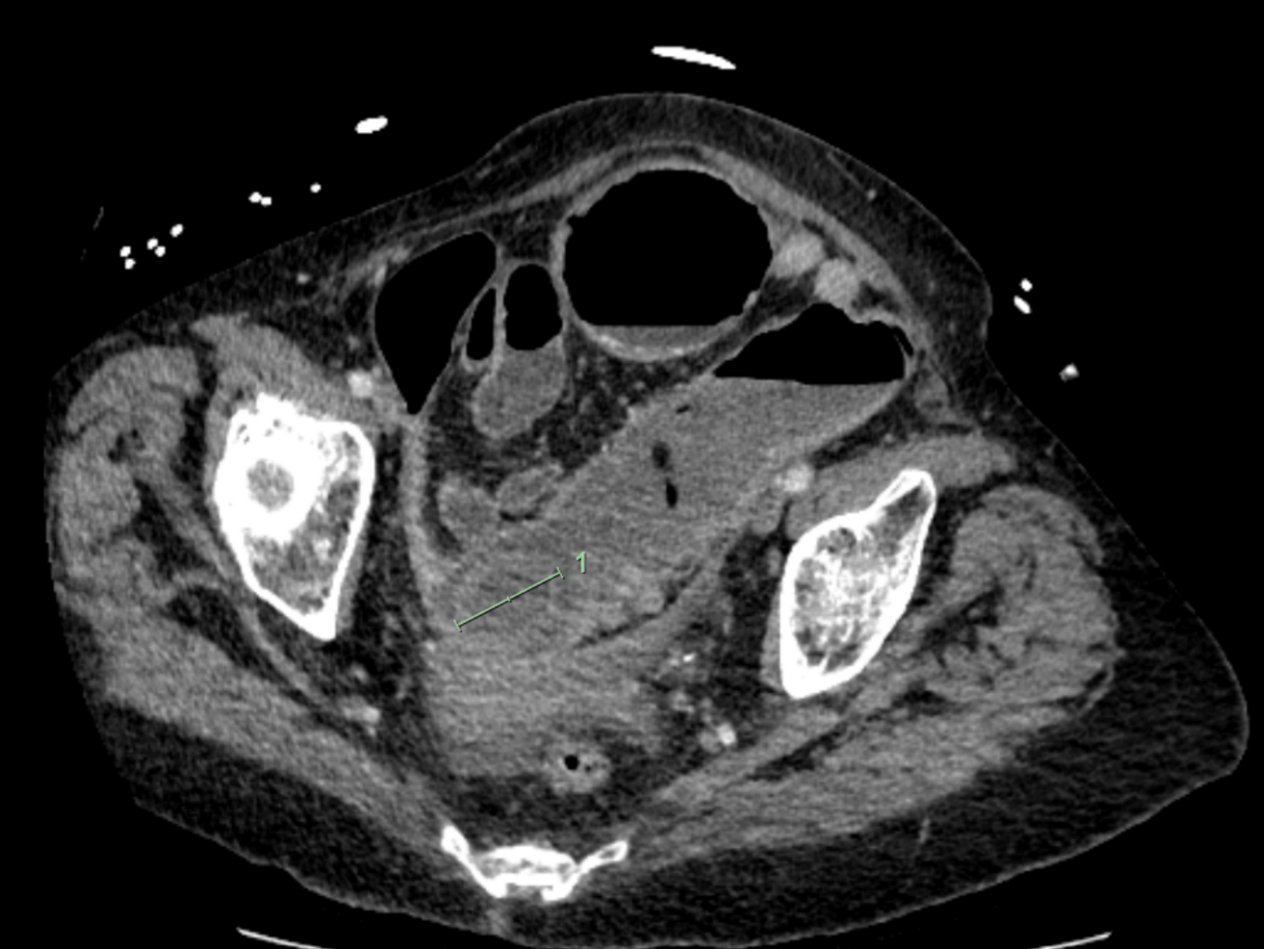
Abdominal CT scan: coronal view showing this impacted gallstone with a dilated large bowel

**Figure 3:**
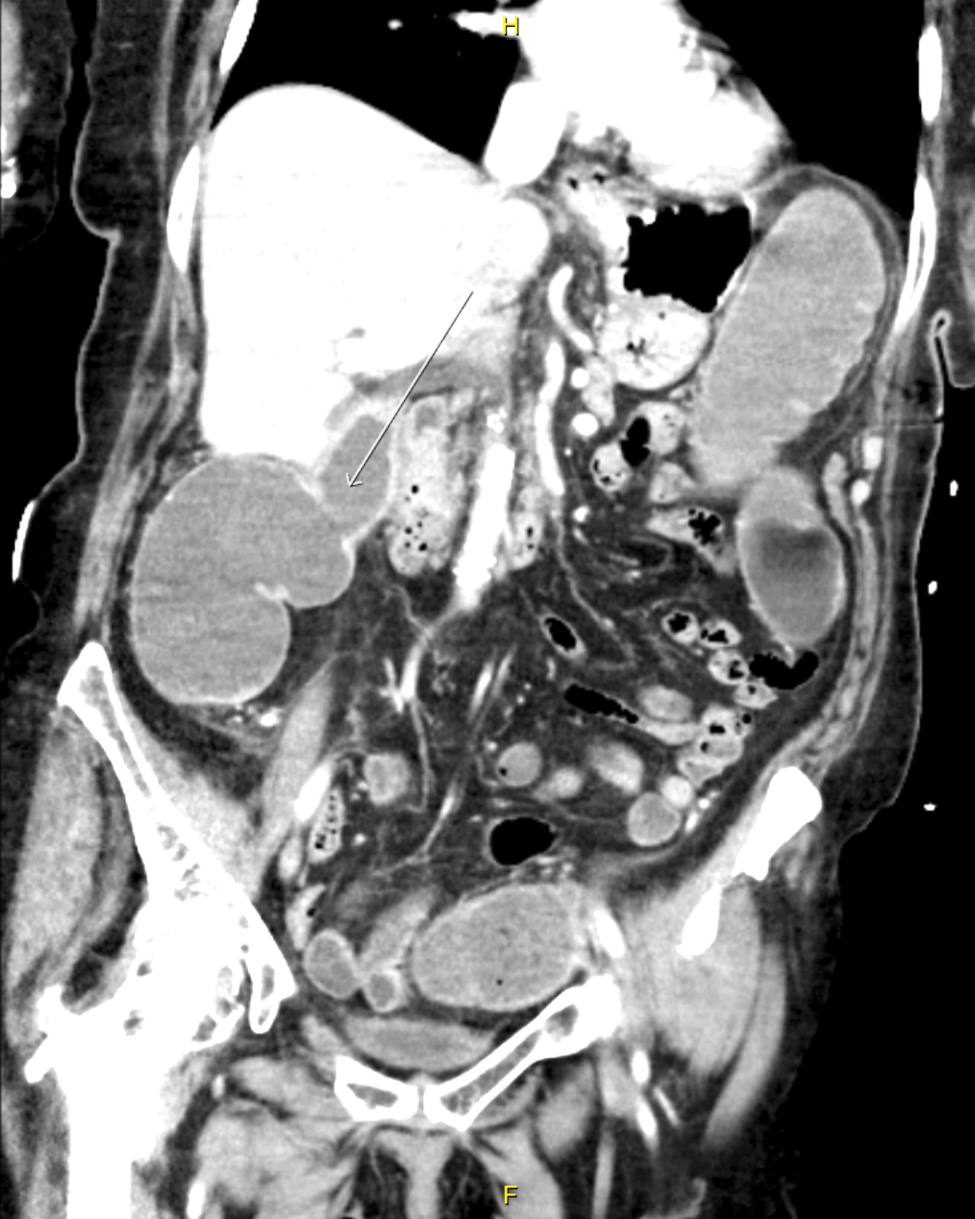
Abdominal CT scan: coronal view showing the cholecysto-colonic fistula connecting the gallbladder to the hepatic flexure (arrow)

**Figure 4:**
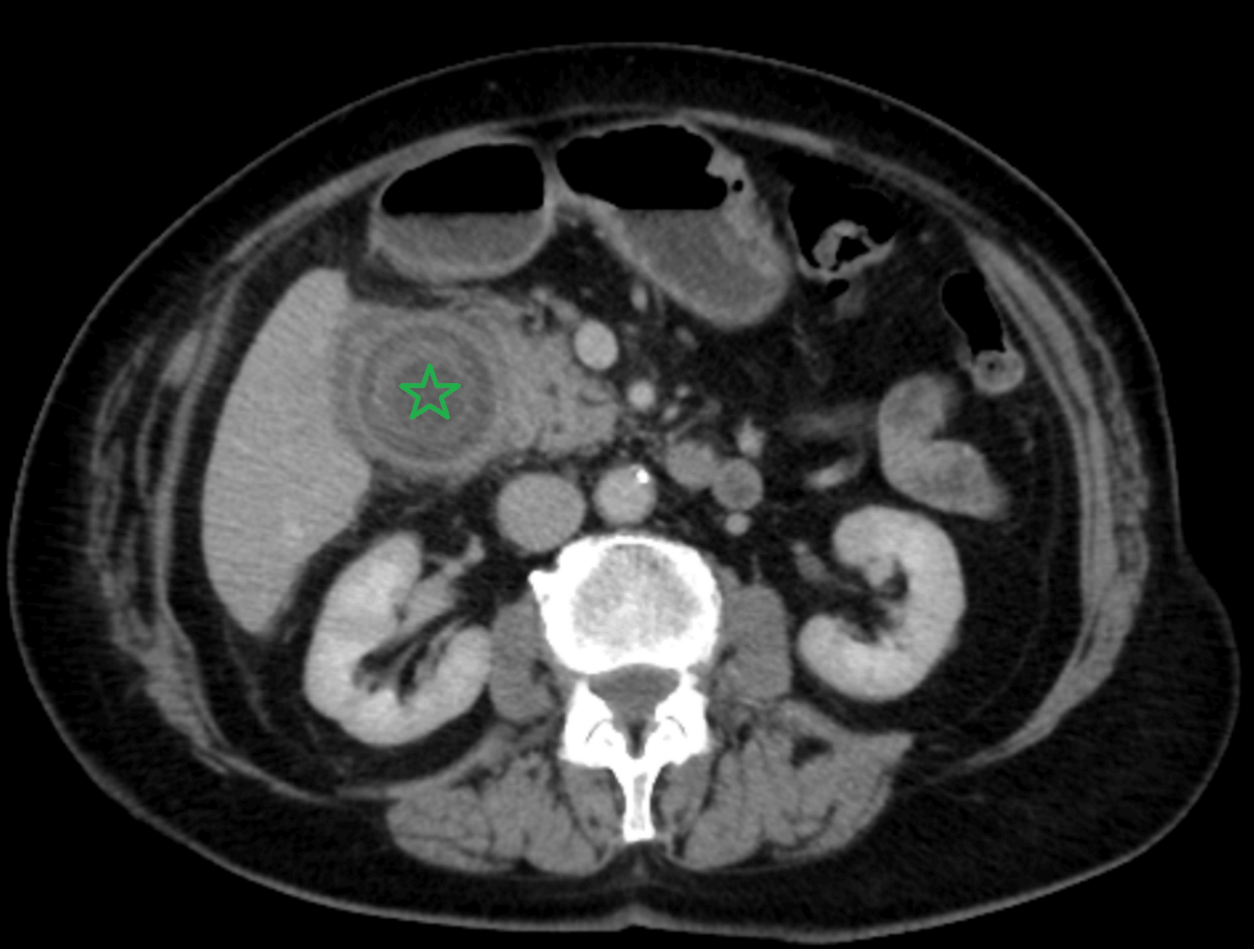
Abdominal CT scan three years ago: axial view showing a 31mm calculus present in the gallbladder neck with chronic cholecystitis. No evidence of a cholecysto-enteric fistula at this stage (green star)

Laboratory results revealed an inflammatory rise (white blood cells: 13.0 × 10^9^/L, C-reactive protein: 110 mg/L). Other results, including liver function and renal function tests, were within normal limits. An initial computerized tomography (CT) scan with intravenous contrast was performed and reported a large bowel obstruction secondary to a 3.5cm sigmoid calculus and a cholecysto-colonic fistula at the level of the proximal transverse colon (Figures 1-3). Of note was her previous CT scan three years ago, which demonstrated chronic cholecystitis and impacted gallstones without any evidence of a cholecysto-enteric fistula (Figure 4).

Treatment

**Figure 5:**
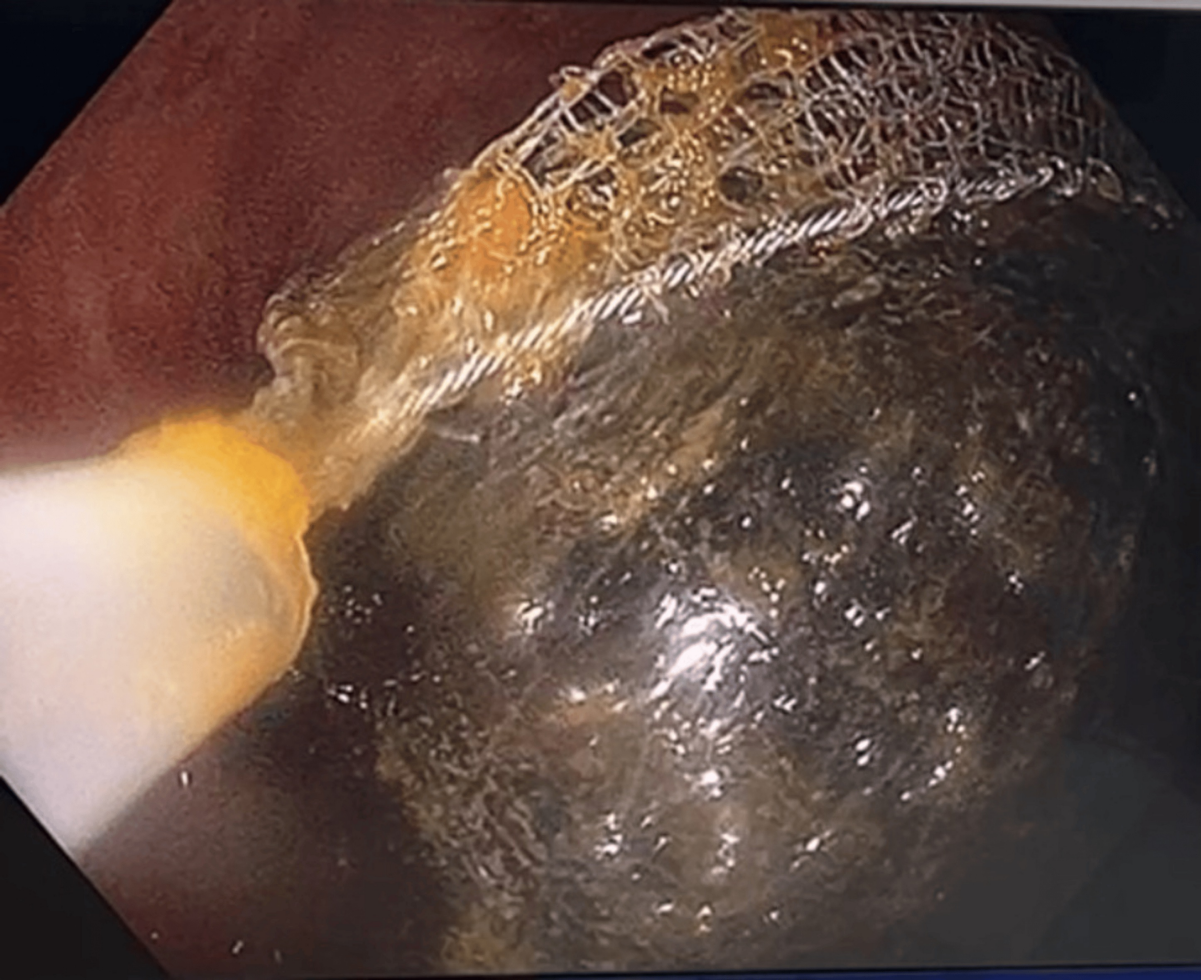
Colonoscopy showing the attempted retrieval of a gallstone using a 3cm endoscopic basket

**Figure 6:**
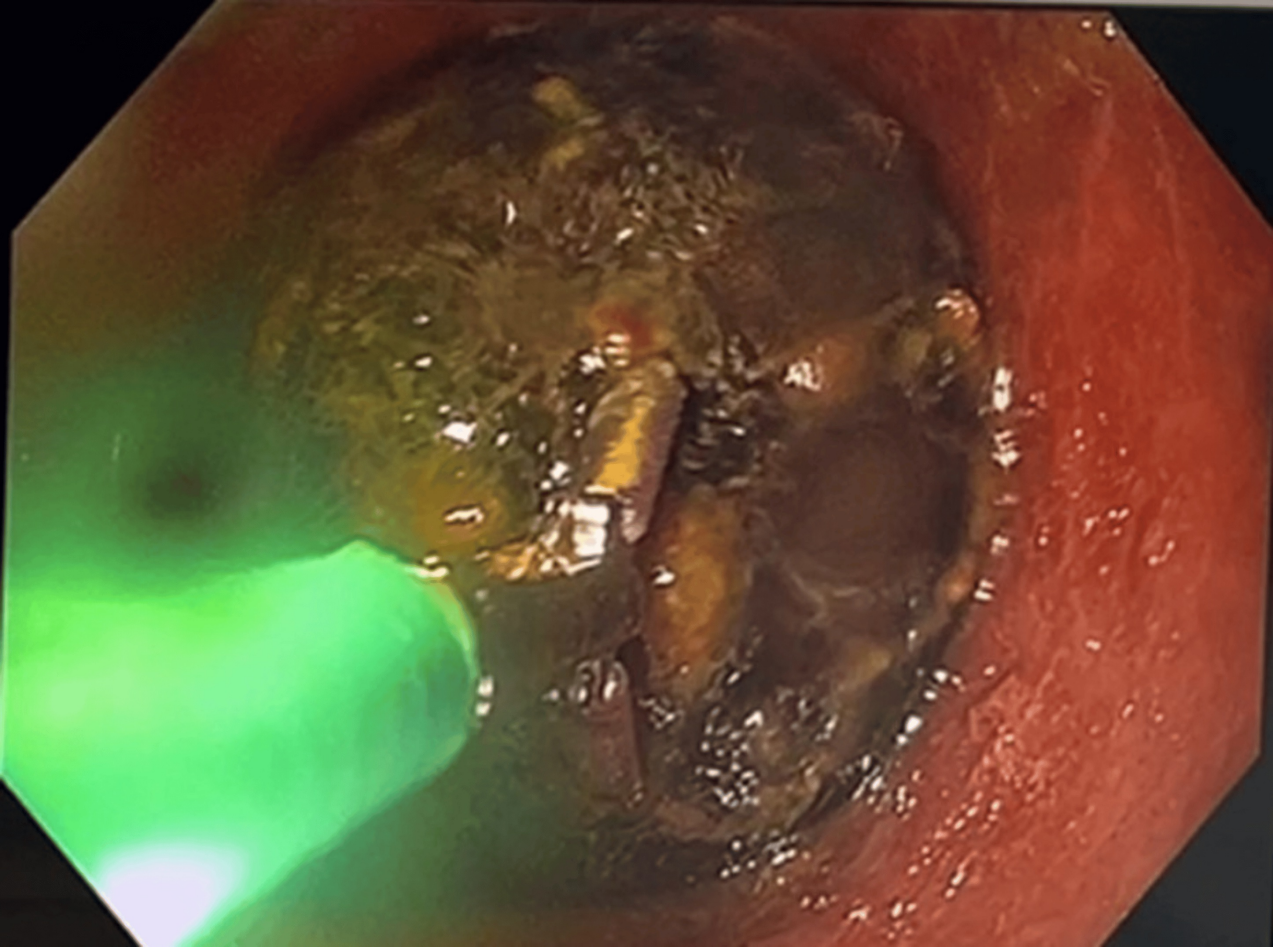
Colonoscopy showing the attempted retrieval of a gallstone using a rat-toothed forceps

**Figure 7:**
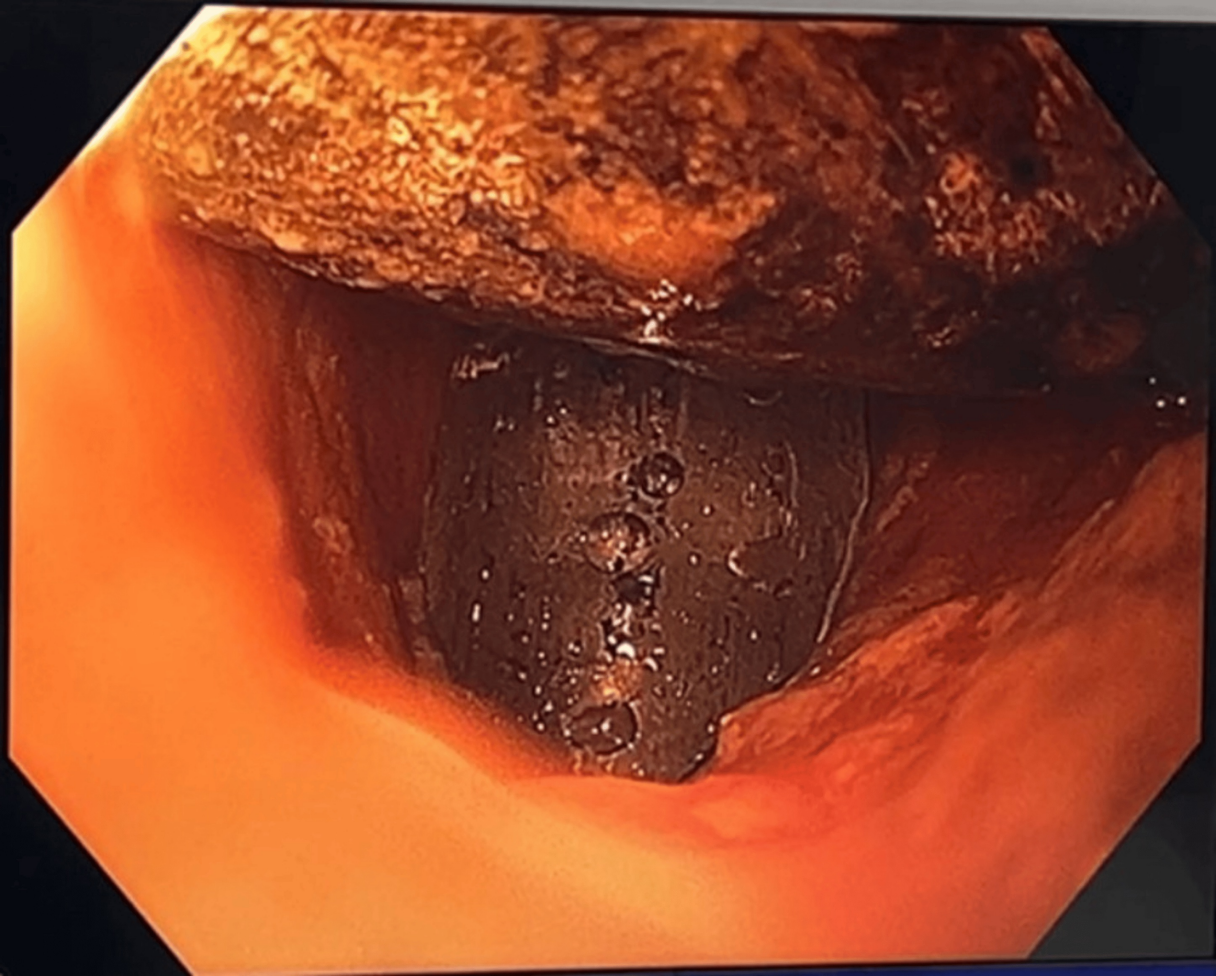
Colonoscopy showing the attempted retrieval of a gallstone using an endoscopic balloon trawl

After initial resuscitation with fluids and intravenous antibiotics, a plan for endoscopic retrieval of the gallstone was made. Colonoscopy revealed a large gallstone impacted at the sigmoid colon, approximately 30cm from the anal verge. Multiple methods were employed in an attempt to retrieve the calculus, including a 3cm endoscopic basket, rat-toothed forceps, and an endoscopic balloon trawl (Figures 5-7).

**Figure 8:**
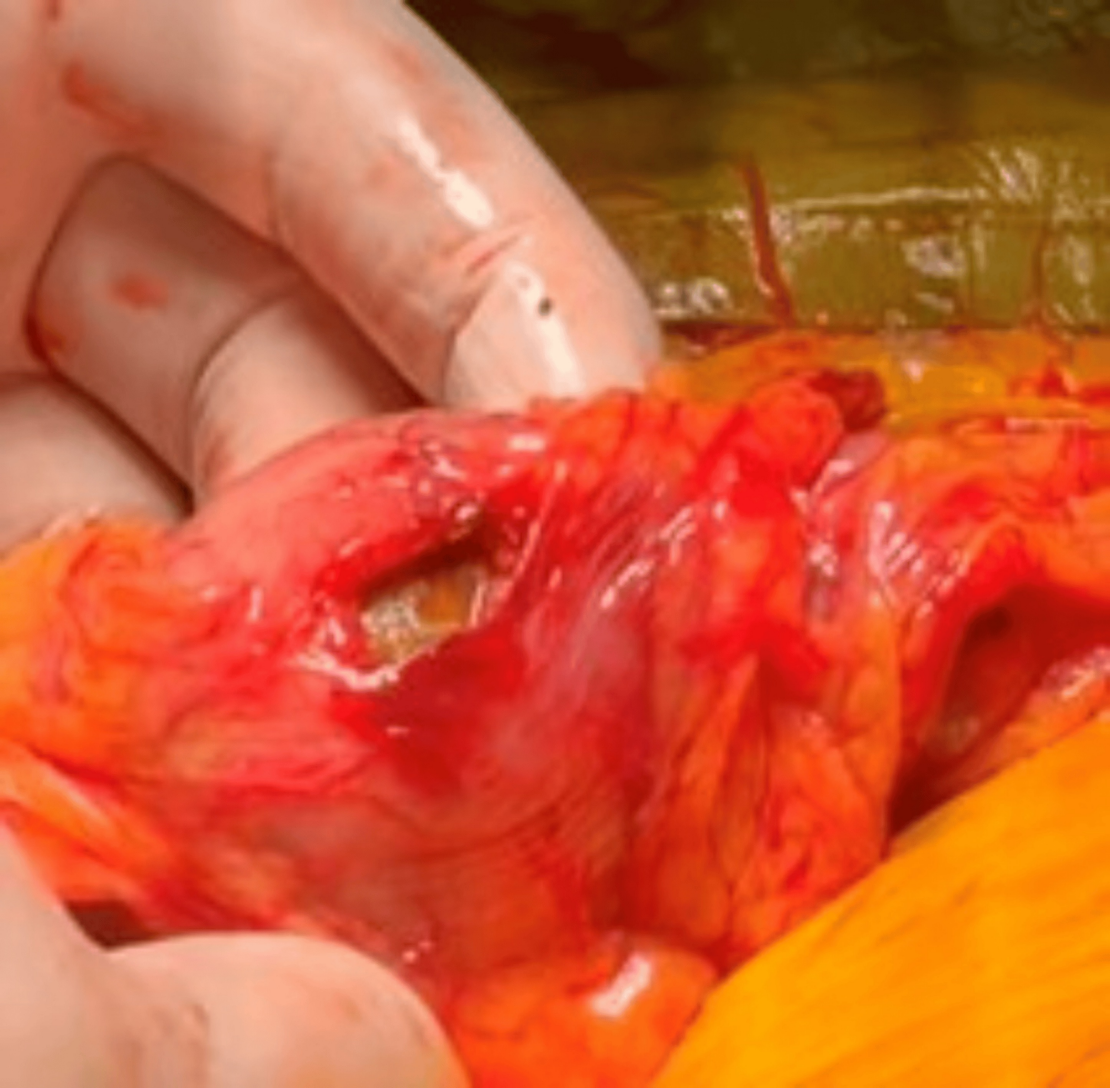
Intraoperative picture showing the pinhole at the sigmoid colon, with a reddened and erythematous colon proximal to the pinhole

**Figure 9:**
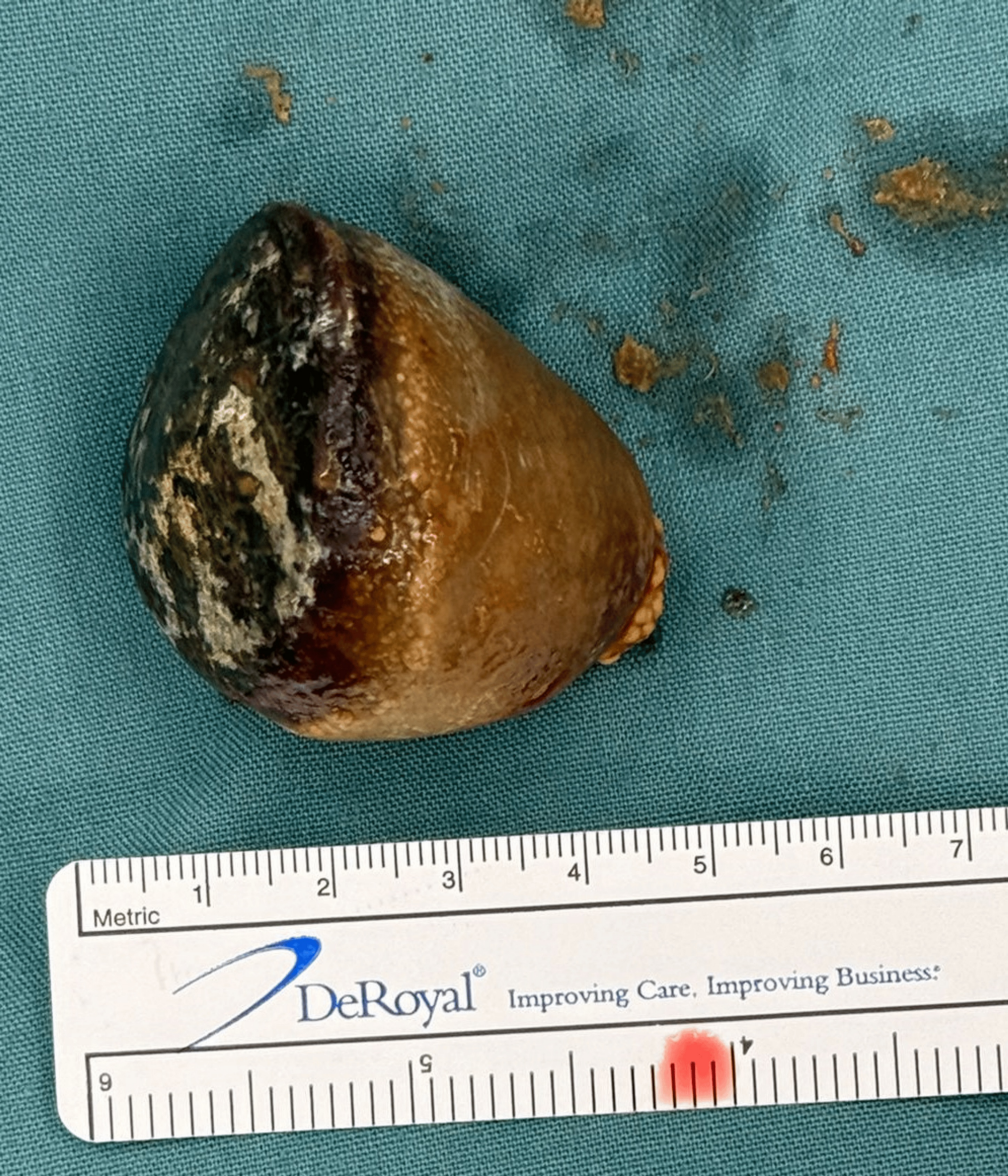
Photograph of the 5cm gallstone

Unfortunately, due to its size and tight lodgment within the colon, endoscopic retrieval was unsuccessful. No diverticular stricture or mass was encountered distal to the impacted point. The patient subsequently underwent an open Hartmann's procedure. The intraoperative findings were significant for a 1cm perforation at the site of impaction, where a 5cm gallstone was extracted (Figures 8, 9). Due to demonstrable sigmoid stenosis over a length of 10cm, the decision was made to resect this segment consistent with Hartmann's procedure.

Outcome and follow-up

The patient had recovered well with the exception of a short period of uncomplicated pancolitis, which improved with intravenous antibiotics. In consideration of the patient's age and the fistula's large diameter, deemed adequate for gallstone passage, a decision was made to defer secondary cholecysto-colonic fistula repair and cholecystectomy following consultation. She was discharged home and remained well after another 26 days of further rehabilitation. Histology reported sigmoid diverticular disease without evidence of malignancy.

## Discussion

This report demonstrates one of the rarest manifestations of gallstone ileus, an undetected sigmoid perforation secondary to a large gallstone impaction in the sigmoid colon. The majority of colonic gallstone ileus occurs in the presence of a biliary-colic fistula, and rarely a biliary-duodenal fistula as a cholelith that passes freely through the terminal ileum and ileocecal valve is unlikely to obstruct the rectosigmoid [5]. This case manifests the 'tumbling phenomenon' with abdominal pain, distention, vomiting, and constipation, prevailing and occurring intermittently as the gallstone lodges and dislodges while passing through the lumen of the bowel. As a result of the phenomenon, this frequently leads to a delayed diagnosis, and deleterious sequelae should be suspected [2].

The primary goal in this case is the prompt relief of the mechanical obstruction. No literature has been reported on the spontaneous passage of gallstones that are larger than 2.5cm [6]. In an effort to circumvent surgery, successful endoscopic retrieval alone has been described in the literature in a few patients [3,6]. However, a 5cm gallstone in a 90-year-old patient and the presence of diverticular disease were significant factors that limited our success and prolonged the anesthesia time. An open Hartmann's procedure involving enterolithotomy was performed to relieve the obstruction and address the pinhole perforation. Given its rarity, no guidelines currently exist for the management of large gallstone ileus in the colon. We propose that endoscopic retrieval should be avoided in elderly patients with a large gallstone ileus of the sigmoid colon. Benefits include reducing anesthetic time and colonic trauma, as the endoscopic procedure may take longer in practice than in theory. Furthermore, the surgeon is more likely to detect a perforation that may be initially occluded by the gallstone secondary to its impaction and subsequent pressure ischemia, which may be missed on imaging, as was this case.

Managing colonic gallstone ileus in a rural hospital presents unique challenges. Rural general surgeons perform procedures with comparable outcomes to metropolitan centers [7]. Moreover, the quality of rurally performed colonoscopies outperforms national standards [8]. However, rural Australians overall exhibit poorer health profiles when compared to metropolitan Australians [7,9]. Geographic remoteness also results in disparities in the quality and accessibility of healthcare, ultimately leading to worse health outcomes and greater all-cause mortality [9]. In addition, geographical spread and low health literacy hinder early recognition of symptoms and delay access to healthcare, increasing risks associated with their conditions. These were important factors at play for our patient, who presented later in her disease course. Another important factor is the limited rural intensive care unit capabilities. As with most rural sites, our center also lacks on-site intensivists, limited and junior medical staff, non-specialist nursing care, and non-specialist anesthetists. These elements risk delaying essential clinical decisions.

Guidelines regarding the timing of cholecystectomy in cholecysto-colonic fistula are not well established [3,6]. Currently, surgical options are primary or delayed cholecystectomy and fistula repair. Augustin et al.'s literature review demonstrated a slightly higher survival rate in the non-cholecystectomy group than the cholecystectomy group, which was statistically not significant (p=0.46) [3]. There is documented mortality from attempted fistula repair and cholecystectomy, which leads to multiple laparotomies for anastomotic leaks or extensive bowel resection [6]. Given the fragility of this patient, there will be more risks for a concurrent cholecystectomy and fistula repair or as a second-stage procedure for this patient.

## Conclusions

Colonic gallstone ileus is rare, and this case highlights a longstanding large calculus in an elderly patient in a rural setting. Patients with large bowel obstruction and evidence of gallstone ileus should be treated with a high index of suspicion for colonic perforation. Endoscopic retrieval should be performed with caution regardless of stone size, with operative management being the mainstay of treatment.

## References

[1] Lassandro F, Romano S, Ragozzino A (2005). Role of helical CT in diagnosis of gallstone ileus and related conditions. AJR Am J Roentgenol.

[2] Reisner RM, Cohen JR (1994). Gallstone ileus: a review of 1001 reported cases. Am Surg.

[3] Augustin G, Bruketa T, Kunjko K, Romić I, Mikuš M, Vrbanić A, Tropea A (2023). Colonic gallstone ileus: a systematic literature review with a diagnostic-therapeutic algorithm. Updates Surg.

[4] (2025). National Medical Workforce Strategy 2021-2031. https://healthcarefunding.specialcommission.nsw.gov.au/assets/Uploads/publications/Exhibits-133/EXHIBIT-H_TAB-H.002.026_MOH.0010.0056.0001.PDF.

[5] Milsom JW, MacKeigan JM (1985). Gallstone obstruction of the colon. Report of two cases and review of management. Dis Colon Rectum.

[6] Farkas N, Kaur V, Shanmuganandan A, Black J, Redon C, Frampton AE, West N (2018). A systematic review of gallstone sigmoid ileus management. Ann Med Surg (Lond).

[7] Paynter JA, Qin KR, Hunter-Smith D, Brennan J, Rozen W (2024). Rural general surgical provision from the perspective of twenty-two rural general surgeons: a thematic analysis. ANZ J Surg.

[8] Watson MM, Watson DC, Maddern GJ, Wichmann MW (2023). Quality of rural colonoscopy outperforms key performance indicators in a multi-centre prospective clinical study. ANZ J Surg.

[9] (2025). Rural and remote health. https://www.aihw.gov.au/reports/rural-remote-australians/rural-and-remote-health.

